# Neu1 Is Released From Activated Microglia, Stimulating Microglial Phagocytosis and Sensitizing Neurons to Glutamate

**DOI:** 10.3389/fncel.2022.917884

**Published:** 2022-05-26

**Authors:** David H. Allendorf, Guy C. Brown

**Affiliations:** Department of Biochemistry, University of Cambridge, Cambridge, United Kingdom

**Keywords:** desialylation, neuraminidase 1, microglia, excitotoxicity, neuroinflammation, neurodegeneration, sialic acid, neurotoxicity

## Abstract

Neuraminidase 1 (Neu1) hydrolyses terminal sialic acid residues from glycoproteins and glycolipids, and is normally located in lysosomes, but can be released onto the surface of activated myeloid cells and microglia. We report that endotoxin/lipopolysaccharide-activated microglia released Neu1 into culture medium, and knockdown of Neu1 in microglia reduced both Neu1 protein and neuraminidase activity in the culture medium. Release of Neu1 was reduced by inhibitors of lysosomal exocytosis, and accompanied by other lysosomal proteins, including protective protein/cathepsin A, known to keep Neu1 active. Extracellular neuraminidase or over-expression of Neu1 increased microglial phagocytosis, while knockdown of Neu1 decreased phagocytosis. Microglial activation caused desialylation of microglial phagocytic receptors Trem2 and MerTK, and increased binding to Trem2 ligand galectin-3. Culture media from activated microglia contained Neu1, and when incubated with neurons induced their desialylation, and increased the neuronal death induced by low levels of glutamate. Direct desialylation of neurons by adding sialidase or inhibiting sialyltransferases also increased glutamate-induced neuronal death. We conclude that activated microglia can release active Neu1, possibly by lysosomal exocytosis, and this can both increase microglial phagocytosis and sensitize neurons to glutamate, thus potentiating neuronal death.

## Introduction

The plasma membrane of mammalian cells is coated with oligosaccharides attached to glycoproteins and glycolipids forming the cell’s glycocalyx. These glycan chains usually terminate in the negatively charged, 9-carbon sugar sialic acid (Wei and Wang, 2019; Klaus et al., 2021). Sialic acids are particularly abundant in the brain and may be attached to gangliosides and glycoproteins *via* α-2,3 or -2,6 glycosidic linkage or to itself *via* α-2,8 linkage forming polysialic acid (Puigdellívol et al., 2020; Klaus et al., 2021). Polysialic acid chains are typically found on neuronal cell adhesion molecule (NCAM) regulating important neuronal functions such as neurite outgrowth (Landmesser et al., 1990), axon pathfinding (Tang et al., 1994) or synaptogenesis (Dityatev et al., 2004). Moreover, recent studies have demonstrated that polysialic acid NCAM (PSA-NCAM) may protect neurons against excitotoxicity by modulating N-methyl-D-aspartate (NMDA) receptors (Hammond et al., 2006; McCall et al., 2013).

The sialic acid residues of glycans may be removed by hydrolytic enzymes called neuraminidases (also known as sialidases), resulting in desialylation of the glycans (Wei and Wang, 2019). Desialylation of receptor glycans can regulate the activity of many different receptors (Wei and Wang, 2019). The main enzyme desialylation glycoproteins is neuraminidase 1 (Neu1), which is highly-expressed in the lysosomes of all mammalian cells (Pshezhetsky and Ashmarina, 2018). In the lysosome, Neu1 function relies on two other proteins, protective protein cathepsin A (PPCA) and β-galactosidase, forming the lysosomal multienzyme complex (Pshezhetsky and Ashmarina, 2001). Interestingly, Neu1 has also been found on the plasma membrane surface of phagocytic immune cells, such as macrophages (Liang et al., 2006). In human macrophage cells, Neu1 is thought be transported together with PPCA in MHCII-positive vesicles to the plasma membrane surface, where it stimulates phagocytosis (Liang et al., 2006).

Microglia are specialized phagocytes of the central nervous system (CNS), and may phagocytose neurons, synapses and dendrites during brain development (Vilalta and Brown, 2018). However, excessive phagocytosis or secretion of proinflammatory factors by microglia may lead to neuronal or synaptic loss, which may contribute to CNS pathologies such as brain ischemia or Alzheimer’s disease (Brown, 2021; Butler et al., 2021). Microglia may be activated by lipopolysaccharide (LPS, also known as endotoxin), lipoteichoic acid (LTA), rotenone or amyloid-β (Aβ), thereby stimulating microglial phagocytosis and toxicity to neurons (Kinsner et al., 2005; Neher et al., 2011; Emmrich et al., 2013; Neniskyte and Brown, 2013). We have previously reported that LPS exposure causes an increased neuraminidase activity on the microglial cell surface and in culture supernatants (Nomura et al., 2017; Allendorf et al., 2020a). This culture supernatant neuraminidase activity was sufficient to desialylate neuronal-like PC12 cells promoting their phagocytosis by microglia (Nomura et al., 2017). We subsequently found that the surface neuraminidase activity of LPS-activated microglia originates from Neu1 (Allendorf et al., 2020b), but it remains unclear whether the released neuraminidase activity is due to Neu1. This is potentially important because this activity is a possible treatment target to prevent neuroinflammation and neurodegeneration, as we found that inhibiting neuraminidases non-specifically was neuroprotective in co-cultures (Nomura et al., 2017; Allendorf et al., 2020a).

Neuraminidase 1 has previously been reported to be released into the extracellular space in platelets (Jansen et al., 2012), but the mechanism/pathway by which Neu1 is released from cells is unclear. Using the microglial cell line Ra-2, Sumida et al. (2015) found that Neu1 was released into the medium bound to extracellular vesicles, called exosomes. The exosome-bound Neu1 appeared to be active as it was able to cleave polysialic acid from microglial NCAM (Sumida et al., 2015). Another possible pathway of Neu1 release is *via* direct fusion of the lysosome with the plasma membrane - a process called lysosomal exocytosis. Lysosomal exocytosis is triggered by a rise in intracellular calcium that induces fusion of lysosomes with the plasma membrane (Tancini et al., 2020). Lysosomal exocytosis is thought to have three main functions, (i) repair of the plasma membrane, (ii) disposal on indigestible lysosomal content, and (iii) secretion of lysosomal proteins (Tancini et al., 2020). However, little is known about microglial lysosomal exocytosis and its function and consequences in the CNS.

Extracellular neuraminidase, released by microglia, could potentially desialylate neurons and thereby change neuronal activities. A neuronal activity potentially regulated by neuraminidases is synaptic activity as there is some evidence that synaptic plasticity (Minami et al., 2017), potassium channel activity (Thornhill et al., 1996) and NMDA receptor activity (Hammond et al., 2006; McCall et al., 2013) are regulated by desialylation of the neuronal surface.

Here we report that LPS induces microglia to release a neuraminidase activity that is reduced by Neu1 knockdown or by inhibition of lysosomal exocytosis. Neu1 protein was present is cell culture supernatant, and eliminated by Neu1 knockdown in the cells. The lysosomal protective protein/cathepsin A (PPCA) was also found in cell culture supernatant in association with Neu1, suggesting that the released Neu1 was active and derived from the lysosomal compartment. Moreover, we report that desialylation of neurons induced by adding either: (i) a sialyltransferase inhibitor, (ii) sialidase or (iii) conditioned media from LPS-treated microglia, sensitizes neurons to glutamate-induced cell death.

## Materials and Methods

### Materials

All chemicals were purchased from Sigma Aldrich and cell culture reagents were from Thermo Fischer, unless indicated otherwise. Lipopolysaccharide (*E. coli* O111:B4), sialidase from *V. cholerea*, sialyltransferase inhibitor 3-Fax-peracetyl Neu5Ac, vacuolin-1, GW4869, FITC-labeled peanut agglutinin were from Sigma. Anti-Neu1 antibody (clone F8) was from Santa Cruz, anti-PPCA antibody was from Proteintech, anti-Lamp1 antibody (clone 1D4B) was from Biolegend, anti-MAP2 antibody (PA5-17646) was from Thermo Fischer, anti-FLAG (M2) was from Sigma. Protein G agarose, protein A/G magnetic beads, siRNAs and LysoTracker Green DND-26 dye were from Thermo Fischer. Gal-3 was a kind gift from Tomas Deierborg.

### BV-2 and Primary Cell Culture

Adherent BV-2 cells were maintained in 10% (v/v) FBS and 1% (v/v) penicillin/streptomycin containing DMEM and passaged every 3 days by trypsinization. Cells were kept at 37 °C and 5% CO2 humidified atmosphere. For treatments BV-2 cells were seeded in DMEM supplemented with 0.5% (v/v) FBS and 1% (v/v) penicillin/streptomycin. The density of live cells in suspension was measured by manual counting using trypan-blue with a Neubauer improved hemocytometer. BV-2 cells were seeded at the appropriate density and left to adhere overnight prior to applying treatments. Neu1 or Trem2-FLAG over-expressing BV-2 microglia were generated as reported previously (Allendorf et al., 2020b).

Due to the overall higher base-line activation of immortalized microglia, we aimed to confirm results from the BV-2 model by using primary microglia. We also used cerebella-derived mixed neuronal-glial co-cultures to study neuronal excitotoxicity. All experiments were performed in accordance with the United Kingdom. Animals (Scientific Procedures) Act (1986) and approved by the Cambridge University local ethical committee. Glial cultures were prepared from postnatal days 5 – 7 rat or mouse cortex. Mixed neuronal-glial co-cultures were from 5 – 7 day old rat or mouse cerebella. Briefly, mouse or rat cerebra were removed from the head of the decapitated animal. Brains were dissected to isolate cerebellum and cortex. Cerebellum and cortex were further dissected in Hank’s balanced salt solution (HBSS) under a light microscope to remove brain meninges. Cortex was dissociated in trypsin- EDTA for 15 min at 37°C, cerebellum was dissociated in Versene (Sigma) for 7 min at 37°C. To make a cell suspension digested tissue was triturated with a pipette. Versene and trypsin were quenched by adding serum containing medium and removed by centrifugation (500 g, 7 min). The attained cell pellet was resuspended in the appropriate culture medium (mixed glia: 10% FBS in DMEM; neuronal-glial co-cultures: 5% FBS, 5% horse serum in DMEM, supplemented with KCl, HEPES, glutamine and glucose). Cortex derived cells were sequentially passed through a 100 and 40 μm cell strainer and seeded in poly-L-lysine coated T-75 flasks. Cerebellum derived cells were passed through a 40 μm cell strainer, counted and seeded in poly-L-lysine coated 24 well plates. 24 h post seeding cellular debris was removed from flasks and plates and medium was exchanged to fresh medium. Primary mouse or rat microglia were isolated 7 DIV by rigorous shaking of the flask. Neuronal-glial co-cultures, made up of approximately 85% neurons and 5% microglia (Bal-Price and Brown, 2001; Neher et al., 2011) were treated 7 DIV.

### Cell Treatments

Primary or BV-2 cells were treated with lipopolysaccharide (LPS) at 100 ng/ml or vehicle (distilled water) for 18 or at 1 μg/ml for 6 hr. Cells were pre-treated with BAPTA-AM at 10 μM or vacuolin-1 at 400 nM for 1hr prior to LPS stimulation. Mixed neuronal-glial co-cultures were desialylated by addition of sialidase at 80 mU/ml for 5 h or by addition of 3-Fax-peracetyl Neu5Ac for 24 h at 100 μM. Sodium glutamate was added at 100 μM for 5-6 h. BV-2 microglia were desialylated by addition of sialidase (from V. *cholerae)* at 200 mU/ml for 3 h.

### Immunoblotting

BV-2 cells or primary mouse microglia were treated with LPS or vehicle for 18 h. Culture supernatants were collected and centrifuged at 500 g to remove detached cells. Supernatants were cleared from debris by further centrifuging at 15.000 g for 20 min and subsequently concentrated 20-fold (BV-2) or 100-fold (primary microglia) using a 10 kDa cut-off filter (Merck Millipore). Samples were heated for 10 min at 95°C in LDS sample buffer (Life Technologies) and DTT (final concentration 50 mM) and loaded onto a precast 4-12% bis-tris NuPage polyacrylamide gel (Life Technologies). Samples from three independent culture preparations were run in duplicate and separated for 45-50 min at 200 V in MOPS SDS running buffer (Life Technologies). Using the NuPage Transblot system (Life Technologies) protein samples were transferred from gel onto a PVDF membrane. Membrane was directly transferred into blocking buffer (5% (w/v) non-fat dry milk in TBS-T) for 1 h and equal loading checked by PonceauS staining. The membrane was incubated over night at 4 °C with either anti-Neu1 (SantaCruz, Clone F8) or anti-PPCA (Proteintech, 15020-1-AP) antibodies at 2 μg/ml. Membranes were washed three times in TBS-T on the following day and incubated with an IRDye800-conjugated anti-rabbit antibody or IRDye680-conjugated anti-mouse antibody (both at 1: 10,000) for 1 h at room temperature. Membrane was washed three times with TBS-T. Detection was carried out using the LICOR system and band intensities were quantified using Image Studio software.

### Cell Staining and Imaging

Live neuronal-glial co-cultures were subjected to nuclear stains Hoechst 33342 (10 μg/ml) and propidium iodide (1 μg/ml) for 15 min at room temperature. Images were taken using the 20x objective of an epifluorescent microscope (Leica DM16000 CS) and propidium iodide positive cells counted using an Image J plugin. Healthy neurons were recognized by their distinct nuclear morphology. Per well four microscopic fields were quantified for a single experiment with *n* = 2 wells per condition.

### Protective Protein/Cathepsin A and Neuraminidase 1 Pull Down Assays

Culture supernatants of LPS or vehicle treated BV-2 microglia were incubated with anti PPCA antibody (Proteintech, 15020-1-AP) or rabbit normal IgG isotype control antibody (Southern Biotech) at 2.5 μg/ml for 4 h at 4°C under constant agitation. Protein G agarose (Thermo Fischer) was washed twice in PBS and added to the supernatants for 2 h at 4°C under agitation. Protein G agarose was washed twice and resuspended in PBS before assaying neuraminidase activity. For Neu1 pull down followed by Western blot analysis, BV-2 culture supernatants were concentrated 20-fold with a 10 kDa cut-off filter (Merck Millipore) and incubated with anti-Neu1 antibody (Clone F8, Santa Cruz) at 3 μg/ml for 4 h at 4°C under agitation. Pull down was performed as described above. Protein G agarose was boiled in LDS sample buffer supplemented with DTT for 10 min at 95°C. SDS-PAGE and Western blot was performed as described in immunoblotting.

### Antibody Staining and PNA/Gal-3 Binding for Flow Cytometry

BV-2 cells were treated with LPS (100 ng/ml) or vehicle for 24 h. Mechanically detached cells were washed several times in PBS supplemented with 5% w/v BSA (Sigma). In subsequent steps, 5% (w/v) BSA in PBS was used as a staining buffer. Cells were incubated with monoclonal rat anti-Lamp1 antibody (Biolegend, clone 1D4B) at 20 μg/ml or normal rat IgG2a isotype control antibody (Invitrogen) for 1 h on ice. After washing the cells three times in staining buffer, secondary Alexa 647-coupled goat anti-rat antibody (Invitrogen) was added for 1 h on ice. After thorough washing the stained cells were directly analyzed by flow cytometry (Accuri C6, BD). Desialylation of live cells was performed as described previously (Allendorf et al., 2020a).

MerTK pulldown and stainings was performed as previously described (Nomura et al., 2017). For receptor desialylation assay TREM2-FLAG construct was transduced using lentivirus into BV-2 microglia as reported previously (Allendorf et al., 2020b). FLAG-expressing or control cells were subjected to LPS (100 ng/ml, 24 h) and subsequently lysed. Lysates from FLAG expressing or control cells were pre-cleared for 1 h with protein A/G magnetic beads (ThermoFischer) and anti-FLAG antibody (M2, Sigma) was added over night at 4°C at 5 μg per 0.5 mg protein. FLAG epitope was pulled down over 4 h with protein A/G magnetic beads. Beads were stained with FITC-labeled peanut agglutinin (15 μg/ml) or TAMRA-labeled Gal-3 (10 μg/ml) for 20 min at room temperature. Mean fluorescence of lectin-stained beads were assessed by flow cytometry.

### Neuraminidase Activity Assay

Endogenous neuraminidase activity in serum-free culture supernatants was assessed by an Amplex Red Neuraminidase Assay Kit (Life Technologies, Carlsbad, CA) following the manufacturer’s instructions. Briefly, 100,000 cells were cultured in phenol red-free DMEM and treated with LPS (100 ng/ml). 18 h post treatment supernatants were taken and spun down at 500 g to remove any detached cells. Supernatants were then subjected to a reagent mix containing 50 μM Amplex Red reagent, 0.1 unit/ml HRP, 2 unit/ml galactose oxidase (from *Dactylium dendroides*) and 250 μg/ml fetuin (from fetal calf serum) in reaction buffer containing 50 mM Tris-HCl (pH 7.2) and 1 mM CaCl_2_ for 30 min at 37°C. Fluorescence was measured on an Optima Plate Reader (BMG Technologies) with 530 nm excitation and 590 nm emission detection.

### Cathepsin Activity Assay

To measure proteolytic activity of cathepsins in culture supernatants, we modified existing protocols using the well-described cathepsin substrate Z-Phe-Arg-4-amido-7-methylcoumarin (Z-Phe-Arg-AMC) (Barrett, 1980). Briefly, 50 μl culture supernatants from 100,000 BV-2 microglia were added to 200 μl of 100 μM Z-Phe-Arg-AMC in pH 4.5 sodium acetate buffer. We used phenol-red free and serum-free DMEM for these experiments. After 30-60 min incubation at 37°C we measured fluorescence of samples in a plate reader at excitation 335 nm and emission 460 nm.

### RNAi in BV-2 Microglia and qPCR

BV-2 cells at 70-80% confluency were subjected to a lipid: siRNA mix containing 3% (v/v) Lipofectamine 3000 (Invitrogen) and 60 pmol of either Neu1-targeting or scrambled siRNA (both Thermo Fischer) in serum free OptiMEM (Gibco). Transfection medium was removed after 3 h incubation at 37°C and replaced by DMEM containing 10% FBS. 24 h post transfection BV- 2 cells were detached, counted and seeded at appropriate density in low serum DMEM. Treatments were routinely applied to cells 24 h post transfection. RNA was extracted with the Qiagen RNease Mini Kit at 24 h post transfection and cDNA was synthesized using SuperScript II Reverse Transcriptase kit (Invitrogen). Neu1 expression was assessed by qPCR using the Platinum SYBR Green qPCR SuperMix (ThermoFischer) and a RotorGene Q machine (Qiagen). Primers for Neu1 were fwd 5′-TTCATCGCCATGAGGAGGTCCA and rev 5′-AAAGGGAATGCCGCTCACTCCA. Data was normalized to a GUSB housekeeper.

### Bead Phagocytosis Assay

Fluorescent and carboxylated 5 μm beads (Spherotech) were added at 0.005% (w/v) for 3 h to BV-2 cells. Media was aspirated, cells washed several times with cold PBS and subsequently detached by trypsinization. Uptake of beads into cells was assessed by flow cytometry (Accuri C6 BD): At least 5,000 cells were analyzed for each treatment replicate. Bead-uptake could be observed in the red fluorescent channel due to the coupling of the beads to the Nile red dye and for each experiment the percentage of cells containing beads was assessed.

### Statistical Analysis

Analysis of data was performed using Graphpad Prism (Vers. 6.0) and data shown represented as a mean of at least *n* = 3 independent experiments ± S.E.M. (standard error of mean). Normality of data and statistical significance was assessed by Shapiro-Wilk and ANOVA followed by Tukey’s or Sidak’s *post hoc* test or by *t*-tests where indicated. *P*-values of *p* ≤ 0.05 are considered significant.

## Results

### Neuraminidase 1 Is Present in BV-2 Culture Supernatants and Extracellular Neuraminidase 1 Enzyme Activity Increases After Lipopolysaccharide Stimulation

**FIGURE 1:**
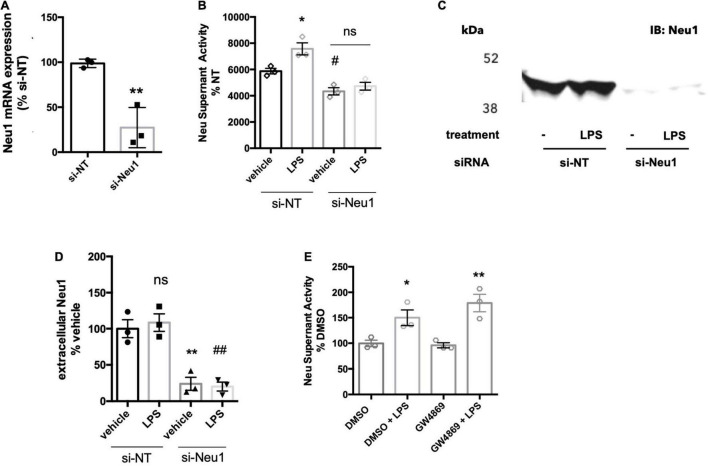
BV-2 microglia release Neu1 into culture supernatants. **(A)** mRNA expression of Neu1 in non-targeting (si-NT) or Neu1-targeting (si-Neu1) siRNA-transfected BV-2 microglia. Knockdown was assessed 24 h after siRNA treatments. Data presented as mean Neu1 expression levels of three independent BV-2 culture preparations (± S.E.M) normalized to the si-NT control. Statistics: unpaired *t*-test, **p < 0.01. **(B)** Supernatant neuraminidase activity assays (pH 7.2) of vehicle or LPS-treated (100 ng/ml, 18 h) BV-2 microglia. 48 h prior to stimulation, BV-2 were transfected with non-targeting or Neu1-targeting siRNA. Data presented as mean neuraminidase activity assays of three independent BV-2 culture preparations (± S.E.M) normalized to the si-NT + vehicle control. Statistics: one-way ANOVA with Tukey’s *post hoc* analysis, *p < 0.05 vs. si-NT + vehicle, #*p* < 0.05 versus si-NT + vehicle, ns: non-significant. **(C)** Western blot of supernatants from si-NT or si-Neu1 transfected BV-2, stimulated with vehicle or LPS (100 ng/ml). Supernatants were concentrated 20-fold with a 10 kDa cut-off filter. Blot representative of three independent experiments. **(D)** Quantification of Western blots of culture supernatants from untreated and LPS-treated BV-2. Data presented as mean Western signal of three independent BV-2 culture preparations (± S.E.M) normalized to untreated control. Statistics: one-way ANOVA with Tukey’s *post hoc* analysis, **p < 0.01 vs. si-NT + vehicle, ##*p* < 0.01 vs. si-NT + vehicle, ns: non-significant. **(E)** Supernatant neuraminidase activity assays (pH 7.2) of vehicle or LPS-treated (100 ng/ml, 18 h) BV-2 microglia. Cells were pretreated with neutral sphingomyelinase inhibitor GW4869 at 20 μM or DMSO control for 1 h before addition of LPS. Data presented as mean neuraminidase activity assays of three independent BV-2 culture preparations (± S.E.M) normalized to DMSO control. Statistics: one-way ANOVA with Tukey’s *post hoc* analysis, *p < 0.05 versus DMSO, **p < 0.01 versus GW4869.

We previously reported that LPS-stimulated BV-2 microglia released a neuraminidase activity into the culture medium (Nomura et al., 2017). We tested whether this activity could be attributed to Neu1 by siRNA-mediated knockdown of Neu1 (with a non-targeting siRNA as control), and verified that the knockdown reduced Neu1 mRNA levels in the cells (Figure 1A). We confirmed here that BV-2 microglia did indeed release a neuraminidase activity into the culture medium (centrifuged to remove any cells or debris, leaving supernatant) that was active at pH 7.2 (i.e., active at extracellular pH), and this activity was significantly increased by LPS treatment (100 ng/ml, 18 h) of the cells (Figure 1B). Knockdown of Neu1 reduced the basal neuraminidase activity in the conditioned culture medium, and prevented LPS from increasing the neuraminidase activity (Figure 1B). Thus, it appears that the neuraminidase activity released by BV-2 microglia treated with LPS is due to Neu1, and that the neuraminidase activity released by BV-2 microglia in the absence of LPS is at least partly due to Neu1.

To test whether Neu1 protein is released by BV-2 microglia, we ran western blots on supernatants of culture medium from BV-2 microglia treated with non-targeting or Neu1-targeting siRNA ± LPS treatment. Using a Neu1-specific antibody, we detected a strong band at approx. 46 kDa for supernatants of microglia ± LPS that was strongly reduced by Neu1 knockdown (Figures 1C,D). The formula molecular weight of mouse Neu1 is 44.5 kDa, not including glycosylation. This confirms that BV-2 microglia release Neu1 into the medium in the presence and absence of LPS.

### Neuraminidase 1 May Be Released by Lysosomal Exocytosis

We investigated the mechanism by which Neu1 was released from microglia. Neu1 has been reported to be released from cells on exosomes (Sumida et al., 2015), so we briefly investigated whether exosome release might be responsible for the release of neuraminidase activity from BV-2 microglia. We tested this by adding the well-characterized inhibitor of exosomal release GW4869, which blocks neutral sphingomyelinase activity. Pre-treatment of BV-2 microglia with the GW4869 at 20 μM did not affect basal or LPS-induced neuraminidase activity compared to a DMSO (solvent) control (Figure 1E). This indicated that exosomes are probably not involved in neuraminidase activity release from BV-2 microglia.

**FIGURE 2:**
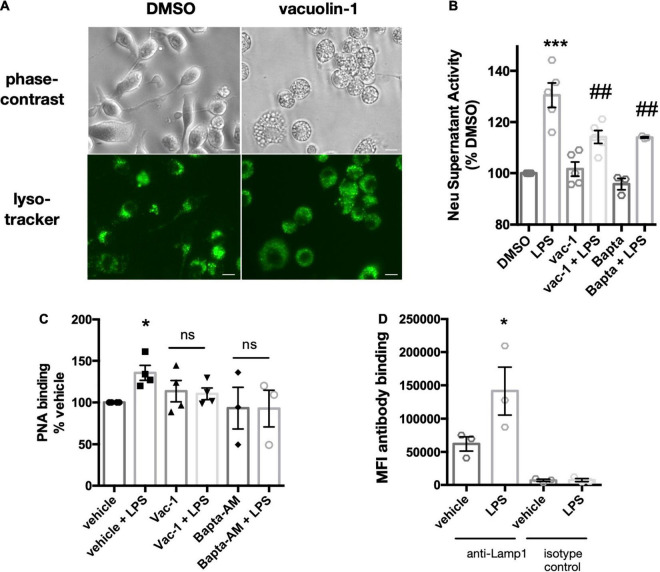
Vacuolin-1 or BAPTA-AM block LPS-induced supernatant neuraminidase activity and surface desialylation. **(A)** Images of DMSO or vacuolin-1 (1 μM, 1 h) treated BV-2 microglia, stained with lysotracker-green. Images acquired with an epifluorescence microscope (20x objective) and representative of three similar experiments. Top panel, bright field. Bottom panel, FITC/488-fluorescence channel. Scale bar – 10 μm **(B)** Supernatant neuraminidase activity assays (pH 7.2) of vehicle or LPS-treated (1 μg/ml, 6 h) BV-2 microglia. Cells were pre-treated for 1 h with inhibitors vacuolin-1 (400 nM) or BAPTA-AM (10 μM). Data presented as mean fluorescence intensities of three independent BV-2 culture preparations (± S.E.M) normalized to DMSO control. Statistical analysis was performed on the original non-normalized data set: one-way ANOVA with Tukey’s *post hoc* analysis, ***p < 0.001 versus DMSO, ##*p* < 0.01 versus LPS. **(C)** Binding of the FITC-conjugated lectin peanut agglutinin (PNA) to BV-2 microglia as measured by flow cytometry. Data presented as fluorescence intensities normalized to DMSO control from *n* = 3 independent experiments. Statistical analysis was performed on the original non-normalized data set: one-way ANOVA with Tukey’s *post hoc* test, *p < 0.05, ns: non-significant. **(D)** Antibody binding (anti-Lamp1 or isotype control) to LPS (100 ng/ml)-stimulated or vehicle treated BV-2 microglia. Binding to live, unfixed cells was assessed by flow cytometry. Data presented as mean fluorescence intensities (MFI) of 5000 events collected from three independent BV-2 culture preparations (± S.E.M). Statistics: one-way ANOVA with Tukey’s *post hoc* analysis, *p < 0.05 versus anti-Lamp1 + vehicle.

Another potential mechanism of Neu1 release is *via* fusion of lysosomes with the plasma membrane, i.e., lysosomal exocytosis, which is calcium-dependent. To test whether the release of extracellular neuraminidase activity by BV-2 microglia was due to lysosomal exocytosis, we used two different inhibitors of lysosomal exocytosis. The small molecule vacuolin-1 inhibits lysosomal fusion with the plasma membrane by inducing fusion of individual lysosomes into larger vacuoles that are unable to fuse with the plasma membrane (Cerny et al., 2004). Treatment of BV-2 microglia with 1 μM vacuolin-1 for 1 h resulted in vacuole formation clearly visible in bright field images (Figure 2A). Furthermore, we observed larger lysosome compartments upon vacuolin-1 treatment visualized by LysoTracker (green channel, Figure 2A). Since we observed considerable cell death after 18 h treatment with vacuolin-1, even at concentrations as low as 100 nM (data not shown), we modified the neuraminidase assay, increasing the concentration of LPS to 1 μg/ml and shortening the treatment time to 6 h. We still observed a significant induction of neuraminidase activity in cell culture supernatants 6 h after LPS stimulation (Figure 2B). Importantly, 1 h pre-treatment with 400 nM vacuolin-1 (vac-1) significantly reduced the LPS-induced neuraminidase activity (Figure 2B). Lysosomal exocytosis is calcium-dependent, and therefore can be inhibited by intracellular calcium chelating agent 1,2-Bis(2-aminophenoxy)ethane-N,N,N′,N′-tetraacetic acid tetrakis-acetoxymethyl ester (BAPTA-AM). Pre-treatment of the cells with 10 μM BAPTA-AM (Bapta) significantly reduced the LPS-induced increase in neuraminidase activity in the cell culture supernatant (Figure 2B). We conclude that the LPS-induced increase in supernatant neuraminidase activity may be mediated by calcium-dependent lysosomal fusion with the plasma membrane.

We then asked whether the LPS-induced supernatant activity was able to effectively desialylate BV-2 microglia. Treatment of BV-2 microglia with 1 μg/ml LPS for 6 h significantly increased binding of FITC-labeled peanut agglutinin (PNA-FITC) as measured by flow cytometry (Figure 2C) - peanut agglutinin only binds to desialylated glycans. BV-2 microglia pre-treatment with vacuolin-1 or BAPTA-AM did not show significant increases in PNA-FITC binding upon LPS stimulation (Figure 2C). We conclude that lysosomal fusion with the plasma membrane may also mediate the LPS-induced surface desialylation of BV-2 microglia.

If lysosomal exocytosis was occurring in the BV-2 microglia, then we might expect to find integral lysosomal proteins on the plasma membrane after LPS stimulation. A well-characterized, integral membrane protein of lysosomes is lysosomal-associated membrane protein 1 (Lamp1), and the exposure of Lamp1 on the plasma membrane is used to monitor lysosomal exocytosis (Tancini et al., 2020). We measured binding of an antibody against the N-terminal (luminal side) of Lamp1 to live BV-2 microglia, and observed increased binding to BV-2 microglia pretreated with LPS for 18 h (Figure 2D). Interestingly, binding of the antibody to untreated cells was also substantially higher than the isotype controls, indicating the presence of Lamp1 also on the surface of unstimulated BV-2 microglia (Figure 2D). This is in accordance with the previous data (Figure 1B) suggesting a basal release of Neu1 into the extracellular environment from BV-2 microglia.

### Neuraminidase 1 Is Released Together With Protective Protein Cathepsin A

**FIGURE 3:**
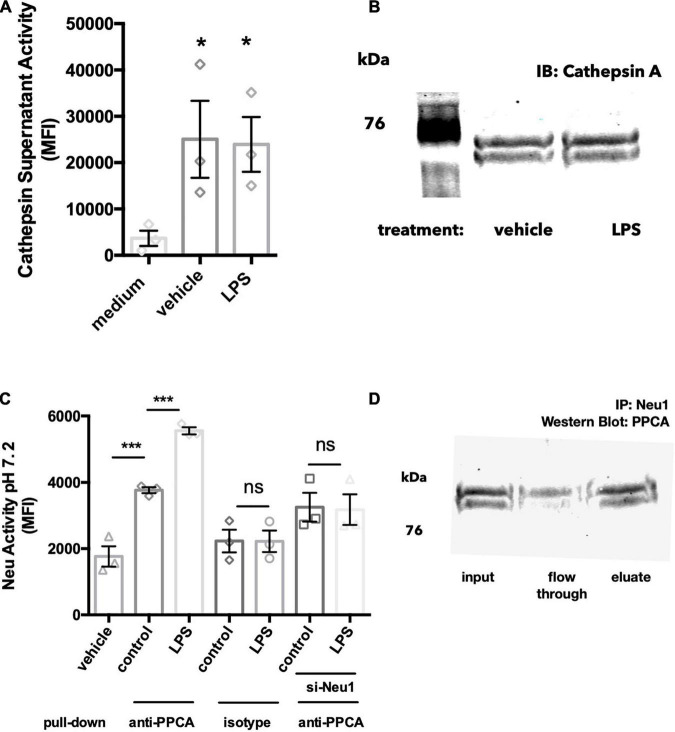
Neu1 is released together with protective protein cathepsin A (PPCA). **(A)** Cathepsin activity assay measured by cleavage of substrate Z-Phe-Arg- 7-amido-4-methylcoumarin (Z-Phe-Arg-AMC) in acidic buffer (pH 4.5). BV-2 cells were treated with vehicle or LPS (100 ng/ml) for 18 h and supernatants were tested for cathepsin activity. Medium indicates a DMEM-only control with no cells added. Data presented as mean fluorescence intensities of at least 3 independent experiments (± S.E.M). Statistics: one-way ANOVA with Tukey’s *post hoc* analysis *p < 0.05 versus medium. **(B)** Western blot of 20-fold concentrated BV-2 culture supernatants, blotting against protective protein cathepsin A (PPCA). Blot representative of 3 similar Western blots. **(C)** Neuraminidase activity assays from protein G agarose pull downs of BV-2 culture supernatants: BV-2 microglia were treated with vehicle or LPS (100 ng/ml) over 18 h. In some experiments BV-2 were pre-treated with si-Neu1-targeting siRNA to reduce Neu1 levels. Culture supernatants were exposed to anti-PPCA or isotype control antibody, followed by protein G agarose pull down. Data presented as mean neuraminidase activity assays of three independent culture preparations (± S.E.M). Statistics: one-way ANOVA with Tukey’s *post hoc* analysis, ***p < 0.001, ns: non-significant. **(D)** Western blot of anti-Neu1 precipitated culture supernatants from LPS-treated BV-2 cells. Blot was probed with an anti-PPCA antibody. Blot representative of three similar experiments.

Within the lysosomes, Neu1 forms a complex with protective protein cathepsin A (PPCA), which protects Neu1 activity (Pshezhetsky and Ashmarina, 2001). Since Neu1 activity was found in the culture medium of BV-2 microglia, we tested whether cathepsin activity was also present in this conditioned medium. We found significant cathepsin activity in this BV-2 conditioned medium, but not in medium that had not been incubated with cells, and LPS had no effect on the cathepsin activity of conditioned medium (Figure 3A). As cathepsin activity is not specific to cathepsin A/PPCA, we also looked for the presence of cathepsin A protein in BV-2 conditioned medium. When concentrating these cell media 20-fold and performing a western blot for PPCA, we found two bands at 65-70 kDa in both vehicle- and LPS-treated conditions (Figure 3B). The formula molecular weight of unglycosylated PPCA is 54.5 kDa, but it is glycolylated (Pshezhetsky and Ashmarina, 2001). This data suggests that Neu1 may be released from BV-2 lysosomes together with its protective protein PPCA.

Since Neu1 and PPCA are known to bind to each other in the lysosome, we next asked whether Neu1 and PPCA are associated extracellularly as well. Firstly, we performed a pull down using an anti-PPCA antibody followed by a neuraminidase activity assay. We were able to detect significant neuraminidase activity in samples precipitated with the PPCA antibody, but not in samples precipitated with an isotype control antibody (Figure 3C). We found a significant increase in neuraminidase activity in anti-PPCA precipitated samples from LPS-stimulated BV-2 cells, and this LPS-induced increase was not observed in supernatant samples from si-Neu1 transfected BV-2 (Figure 3C). Overall, this data indicates that extracellular PPCA is associated with a neuraminidase, probably Neu1.

We further confirmed this by performing western blot analysis of the anti-Neu1 precipitated supernatants of LPS-stimulated BV-2 microglia. We observed a band corresponding to the size of PPCA in the eluted fraction (Figure 3D). This further supports the idea that Neu1 and PPCA indeed form a complex in BV-2 culture supernatants.

### Primary Microglia Also Release Neuraminidase 1

**FIGURE 4:**
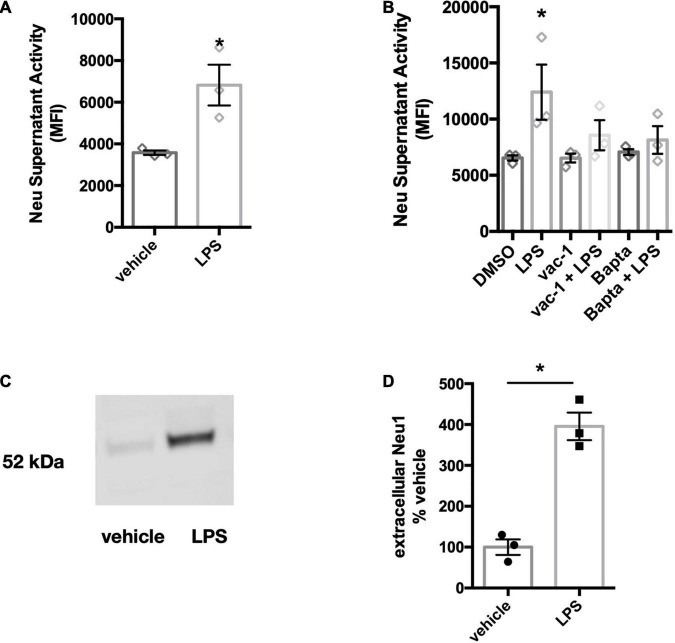
Primary microglia treated with LPS also release neuraminidase activity and Neu1 protein. **(A)** Supernatant neuraminidase activity assays (pH 7.2) of vehicle or LPS-stimulated primary rat microglia (100 ng/ml LPS, 18 h). Data presented as mean neuraminidase activity assays of three independent culture preparations (± S.E.M). Statistics: unpaired *t*-test, *p < 0.05. **(B)** Supernatant neuraminidase activity assays (pH 7.2) of vehicle or LPS-treated (1 μg/ml, 6 h) primary rat microglia. Cells were pre-treated for 1 h with inhibitors vacuolin-1 (400 nM) or BAPTA-AM (10 μM). Data presented as mean fluorescence intensities of three independent cell culture preparations (± S.E.M). Statistics: one-way ANOVA with Tukey’s *post hoc* analysis, *p < 0.05 versus DMSO. **(C)** Western blot of concentrated culture supernatants from primary mouse microglia, stimulated with 100 ng/ml LPS for 18 h. Blot was probed against Neu1 protein. Blot is representative of 3 similar experiments and quantified in **(D)**. Statistical analysis was performed by paired *t*-test, *p < 0.05.

As BV-2 cells are an immortal cell line, we also tested whether the release of neuraminidase activity was observable in primary microglia. Cortical rat microglia were treated with LPS (100 ng/ml) for 18 h and supernatants of the conditioned culture media were screened for neuraminidase activity at neutral pH. We observed a neuraminidase activity in these media, and this activity was significantly increased by LPS treatment of the BV-2 microglia (Figure 4A). Similar to BV-2 microglia, the release of neuraminidase activity into culture supernatants was significantly reduced by the inhibitors of lysosomal exocytosis, vacuolin-1 and BAPTA- AM (Figure 4B).

Since the anti-mouse Neu1 antibody did not cross-react with rat-derived Neu1 protein, we tested whether Neu1 protein would be released in LPS-stimulated cultures of primary mouse microglia. We treated primary microglial cultures with 100 ng/ml LPS for 18 h and concentrated supernatants 100-fold. We observed a band corresponding to the size of Neu1 protein in these conditioned media, and LPS increased the amount of Neu1 protein (Figures 4C,D). This confirms that primary microglia release Neu1 upon LPS stimulation.

### Neuraminidase 1 Increases Microglial Phagocytosis

**FIGURE 5:**
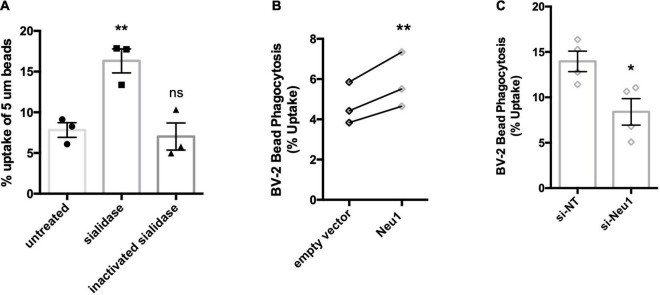
Neu1 regulates microglial phagocytosis. Phagocytosis of 5 μm beads by BV-2 microglia as measured by flow cytometry. **(A)** Assessment of bead uptake 3 h after treatment with sialidase (200 mU/ml). Heat-inactivated sialidase was included as a control. Data presented as mean uptake measured in 3 independent experiments with error bars representing S.E.M. Statistical analysis was performed by one-way ANOVA followed by Tukey’s *post hoc*, **p < 0.01, ns: non-significant. **(B)** Phagocytosis was assayed 48 h post non-targeting (si-NT) or Neu1-targeting (si-Neu1) siRNA transfection. Data presented as mean uptake measured in 4 independent experiments with error bars representing S.E.M. Statistical analysis was performed by unpaired t-test, *p < 0.05. **(C)** Phagocytic capacity of lentiviral transduced, Neu1-overexpressing cells was assessed. Data presented as individual uptake measurements from 3 independent experiments. Statistical analysis was performed by paired (B) *t*-tests, **p < 0.01.

Having established that LPS-activated microglia release a Neu1 neuraminidase activity, we then investigated the consequences, first for microglial phagocytosis, and subsequently for neuronal toxicity. We have previously reported that treatment of microglia with neuraminidase increases microglial phagocytosis (Allendorf et al., 2020a), and we confirmed this here (Figure 5A), thus extracellular neuraminidase is sufficient to stimulate phagocytosis. We then tested whether lentiviral overexpression of Neu1 in BV-2 microglia affected phagocytosis, and found that Neu1 overexpression stimulated phagocytosis of beads (Figure 5B). We also tested whether siRNA-mediated knockdown of Neu1 in BV-2 microglia affected phagocytosis, and found that Neu1 knockdown inhibited phagocytosis of beads (Figure 5C). Thus, Neu1 can regulate microglial phagocytosis.

### Lipopolysaccharide Activation of Microglia Induces Desialylation of Trem2 and MerTK

**FIGURE 6:**
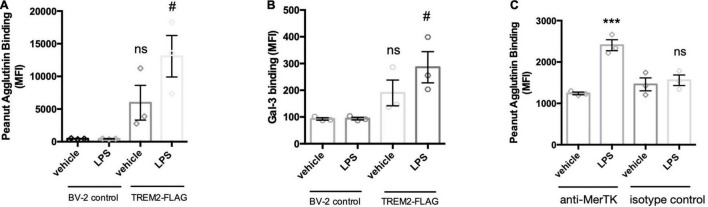
LPS-mediated activation of BV-2 induces removal of sialic acid residues from TREM2 and MerTK receptor. TREM2-FLAG or control BV-2 were treated with vehicle or LPS (100 ng/ml 18 h). Cells were lysed and FLAG-tagged TREM2 captured by anti-FLAG antibody. MerTK was captured on the beads by an anti-MerTK antibody (or appropriate isotype control antibody to control for non-specific binding). Antibodies were captured by protein A/G magnetic beads and subjected to PNA-FITC or Gal-3-TAMRA lectins. **(A)** Binding of PNA-FITC or **(B)** Gal-3-TAMRA to FLAG pull downs from LPS-treated control or TREM2-FLAG-expressing cells. Data presented as mean fluorescence intensities ± S.E.M. Statistics: paired t-tests, #*p* < 0.05 versus TREM2-FLAG + vehicle, ns: non-significant versus BV-2 control + vehicle. **(C)** Binding of PNA-FITC to MerTK pull-downs from LPS- or vehicle treated BV-2 microglia. Data presented as mean fluorescence intensities ± S.E.M. Statistics: one-way ANOVA followed by Dunnett’s multiple comparison test, ***p < 0.001.

LPS activation of microglia releases a sialidase activity, which can increase microglial phagocytosis. In relation to the mechanism of this increased phagocytosis, we were interested in whether the phagocytic receptors of microglia would be desialylated, as it is known that desialylation can regulate receptor activities (Wei and Wang, 2019). We previously developed a method to detect desialylation of glycosylated receptors using an antibody-capture assay (Allendorf et al., 2020b). The flow cytometry-based assay measures binding of FITC-labeled peanut agglutinin (PNA) to receptors that were previously captured by an appropriate antibody. Peanut agglutinin and galectin-3 exclusively bind to terminal galactose residues, and this binding is blocked by terminal sialic acid residues, but the binding is enables by desialylation. Here we use either an anti-FLAG or an anti-MerTK antibody to pull-down Trem2-FLAG or MerTK expressed in BV-2 cells. PNA-FITC and galectin-3 (Gal-3, TAMRA-labeled) bound significantly more to Trem2-FLAG pull-downs from cells that were pre-treated with LPS (Figures 6A,B). LPS-treatment did not affect PNA-or Gal-3 binding to pull-downs from control (non-FLAG expressing) BV-2 (Figure A,B). This indicated that LPS treatment indeed induced the removal of sialic acid residues from the Trem2 receptor, and enabled binding of one of Trem2’s ligands: Gal-3.

The sialylation state of MerTK was assessed by pull-down with an anti-MerTK antibody of lysates from LPS- or vehicle-treated BV-2 microglia. An isotype antibody was used to control for non-specific binding to the antibody or the protein A/G-coated beads. Interestingly, LPS-treatment of the BV-2 microglia significantly increased PNA binding to pull-downs from MerTK-immunoprecipitated lysates (Figure 6C). This increase PNA-binding was not observed for pull-down samples from isotype control treated lysates. Increased binding of Gal-3 to MerTK after microglial activation was demonstrated previously by us (Nomura et al., 2017). Overall, these data indicate that LPS activation of microglia results in desialylation of two phagocytic receptors of microglia: Trem2 and MerTK, enabling them to bind Gal-3, a ligand of both Trem2 and MerTK (Nomura et al., 2017; Boza-Serrano et al., 2019), potentially enabling phagocytosis *via* these receptors and Gal-3.

### Desialylation of Neurons Increases Their Sensitivity to Glutamate-Induced Death

**FIGURE 7:**
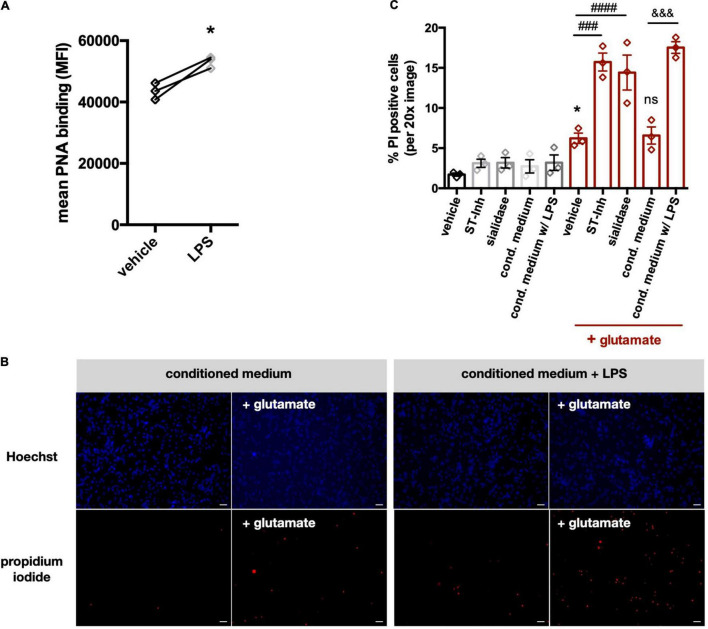
Conditioned media from LPS-stimulated microglia induce desialylation in neuronal-glial co-cultures and sensitize neurons to glutamate-induced excitotoxicity. **(A)** Peanut agglutinin binding to neuronal-glial co-cultures that were pretreated with (i) conditioned media from primary glial cultures (vehicle) or (ii) conditioned media from LPS-stimulated (i.e., 100 ng/ml, 18 h) primary glial cultures (LPS). Binding was measured by flow cytometry on live cells and data presented as mean fluorescent intensities of the PNA-FITC dye. Statistics: paired *t*-test, *p < 0.05. **(B)** Neuronal-glial co-cultures were treated for 1 h with conditioned medium from unstimulated microglia or conditioned medium from LPS-stimulated microglia. Sodium glutamate (100 μM) was then added for 5 h and propidium iodide (PI) positive neurons were counted. Representative images of Hoechst and propidium iodide stained neuronal-glial co-cultures after treatment with conditioned media (with or without LPS) at 20x magnification. Scale bar − 75 μm. **(C)** Quantification of PI positive cells in neuronal-glial cultures, which where desialylated with either sialidase, sialyltransferase inhibitor (ST-Inh) or conditioned medium (cond. medium) from LPS-treated primary rat microglia, and then stimulated with 100 μM glutamate. Data represents mean PI positive cells (± S.E.M) of at least 6 images from 3 independent neuronal-glial cultures. *p < 0.05 vs. vehicle + no glutamate; ###*p* < 0.001, ####*p* < 0.0001 and ns (non-significant) versus vehicle + glutamate: &&& *p* < 0.001 versus conditioned medium + glutamate.

Extracellular sialidase, released by microglia, could potentially desialylate neurons and thereby change neuronal activities, for example by regulating NMDA-type glutamate receptors (Hammond et al., 2006; McCall et al., 2013). Thus, we investigated whether the neuraminidase released by microglia affected neuronal sialylation and viability. We found that when the conditioned media from cortical rat microglia was added to neuronal-glial co-cultures (derived from rat cerebella and containing 85% neurons), it induced increased peanut agglutinin (PNA) binding to the cells, indicating surface desialylation of the cells (Figure 7A), but had no significant effect on neuronal viability when added on its own (Figure 7C). However, when the conditioned media was added together with a low dose (100 μM) of glutamate, the conditioned media substantially increased neuronal death (Figures 7B,C). To test whether an extracellular sialidase alone could sensitize to glutamate, we added sialidase ± glutamate to the cultures, and found that sialidase alone had no significant effect on neuronal death, but it sensitized to glutamate-induced death (Figure 7C). We have previously shown that neurons can also be desialylated using a pan-sialyltransferase inhibitor (Allendorf et al., 2020a), so we tested here whether this neuronal desialylation sensitized to glutamate-induced death. Indeed, as with the other treatments, the sialyltransferase inhibitor had no significant effect on neuronal death alone, but it sensitized to glutamate-induced death (Figure 7C). Thus, sialyltransferase inhibitor, sialidase or microglia-conditioned media containing sialidase, all sensitize neurons to glutamate-induced death.

## Discussion

This study found evidence that microglia release a neuraminidase activity, which is largely due to Neu1. We detected Neu1 protein and neuraminidase activity in supernatants of unstimulated BV-2 cells, and LPS increased the released neuraminidase activity. Neu1 knockdown reduced the released neuraminidase activity of both unstimulated and LPS-stimulated BV-2 cells, but after Neu1 knockdown, LPS no longer increased neuraminidase release. This indicates that the LPS-induced neuraminidase activity was due to Neu1, and that Neu1 also contributes to neuraminidase activity released by unstimulated BV-2 cells. In primary microglia, we also observed an LPS-induced release of neuraminidase activity and Neu1 protein, which was greater than that in BV-2 microglia, consistent with BV-2 being basally activated.

Since Neu1 is a lysosome-resident protein we investigated whether exocytosis of lysosomes was responsible for Neu1 release into culture supernatants. Using an inhibitor of lysosomal exocytosis, vacuolin-1, we observed a reduction in extracellular Neu activity with both BV-2 and primary rat microglia, indicating that lysosomal exocytosis may be involved in the release of neuraminidase activity. Vacuolin-1 has been described as efficiently promoting fusion of mature lysosomes without affecting other intracellular membranes (Cerny et al., 2004). Nevertheless, we cannot rule out that the detected Neu1 protein originated from other compartments such as the Golgi.

Lysosomal exocytosis is also inhibited by the intracellular calcium chelator BAPTA-AM, which blocks calcium-induced synaptotagmin-mediated release of vesicles (Chapman, 2002). We found that BAPTA-AM blocked LPS-induced release of extracellular neuraminidase in BV-2 and primary rat microglia. This supports a role for lysosomal exocytosis in this release. However, exosome release may also be dependent on calcium. On the other hand, we found that a well-characterized inhibitor of exosome formation and release, GW4869 (Menck et al., 2017), did not prevent neuraminidase release from BV-2 microglia. We cannot exclude a role for exosomes based on the use of an inhibitor alone, but a recent proteomic analyses of secreted exosomes from BV-2 cells did not detect Neu1 or any other neuraminidases (Yang et al., 2018). This makes exosomes an unlikely source of Neu1 in BV-2 microglial culture supernatants. Liang et al. (2006) have suggested that Neu1 (together with PPCA) reaches the surface of macrophages in vesicles budding off the lysosomes and fusing with the plasma membrane, and we can not rule out this possibility in microglia, but Liang et al. (2006) also did not rule out the possibility that Neu1 reaches the surface of macrophages by lysosomal exocytosis.

To further investigate whether LPS was inducing lysosomal exocytosis, we tested for the presence of lysosome-associated membrane protein 1 (Lamp-1) on the cell surface, as this is a reliable indicator for the exocytosis of mature lysosomes (Tancini et al., 2020). Indeed, we found the anti-Lamp1 antibody bound to unstimulated BV-2 and observed increased binding to LPS-stimulated cells. Lysosomes contain cathepsins, and the release of cathepsin activity from cells is used as a measure of lysosomal exocytosis (Tancini et al., 2020). We found that BV-2 cells indeed released a cathepsin activity that cleaved the cathepsin substrate Z-Phe-Arg-AMC. This activity was not increased by LPS which may be due to limitations of the cathepsin assay, such as the high variation in signal across different cell culture supernatant preparations. One cathepsin, normally found in lysosomes, is protective protein cathepsin A (PPCA), and we were able to detected PPCA in BV-2 culture supernatants. Importantly, lysosomal PPCA is normally found in complex with Neu1, and stabilizes Neu1 activity (Pshezhetsky and Ashmarina, 2001). Since we found both proteins in culture supernatants, we tested if they associate extracellularly. Indeed, our data indicates that PPCA and Neu1 also form a complex in culture supernatants, which further supports the hypothesis that these proteins are released *via* lysosomal exocytosis, and the Neu1 remains active.

A microglial released neuraminidase activity might regulate a variety of cell functions extracellularly. As extracellular neuraminidase can stimulate microglial phagocytosis (Allendorf et al., 2020a), we tested whether Neu1 expression regulated microglial phagocytosis. We found that Neu1 knockdown reduced microglial phagocytosis, while Neu1 overexpression increased microglial phagocytosis. Thus, Neu1 regulates microglial phagocytosis, and as activated microglia release Neu1, this might be one means by which activated microglia increase phagocytosis.

Desialylation might increase phagocytosis as a result of desialylation of phagocytic receptors, so we tested whether two key phagocytic receptors, Trem2 and MerTK, were desialylated in LPS-activated microglia. We found that LPS induced increased desialylation of Trem2 and MerTK, as indicated by binding of peanut agglutinin and galectin-3. We don’t know whether this is mediated by Neu1 and whether desialylation increases the receptor activity of these receptors, as it does for other receptors (Wei and Wang, 2019), but as galectin-3 is a functional ligand for both receptors (Nomura et al., 2017; Boza-Serrano et al., 2019), this suggests that desialylation enables receptor activation and function via galectin-3.

A neuraminidase activity released from microglia might also desialylate neurons, and we confirmed this occurred with primary neurons. Desialylation of neurons might affect a variety of neuronal functions, such as NMDA receptor activity (Hammond et al., 2006; McCall et al., 2013). So, we tested whether desialylation of neurons affected the sensitivity of neurons to glutamate-induced death. We found that neuronal desialylation, induced by added neuraminidase, sialylation inhibitors or microglia-conditioned media, caused a large increase in glutamate-induced neuronal death. It should be noted that, because of the quantities of cells involved, microglia-conditioned media were derived from cortical microglia and were added to cultures derived from the cerebellum, and the microglia and neurons from these brain regions may differ. Overall, this data indicates that neuronal desialylation may sensitize to excitotoxicity. Potential mechanisms for this might be: desialylation of NCAM-PSA known to regulate the sensitivity of the NMDA receptor to glutamate (Hammond et al., 2006; McCall et al., 2013), or desialylation of potassium channels known to regulate neuronal excitability (Thornhill et al., 1996). Whatever the mechanism, these results suggest that inflamed microglia may sensitize neurons to glutamate *via* released neuraminidase. In a physiological context, this might enhance excitatory signaling, but in a pathological context, this might enhance excitotoxicity, consistent with previous finding that activated glia induce neuronal death *via* excitotoxicity (Bal-Price and Brown, 2001). This suggests that extracellular Neu1 might be a potential treatment target to prevent neuroinflammatory damage to neurons.

## Data Availability Statement

The original contributions presented in the study are included in the article/supplementary material, further inquiries can be directed to the corresponding author/s.

## Ethics Statement

The animal study was reviewed and approved by The Animal Welfare and Ethical Review Body – University of Cambridge.

## Author Contributions

DA designed and performed the experiments and data analysis. DA and GB conceived the project and wrote the manuscript. Both authors contributed to the article and approved the submitted version.

## Conflict of Interest

The authors declare that the research was conducted in the absence of any commercial or financial relationships that could be construed as a potential conflict of interest.

## Publisher’s Note

All claims expressed in this article are solely those of the authors and do not necessarily represent those of their affiliated organizations, or those of the publisher, the editors and the reviewers. Any product that may be evaluated in this article, or claim that may be made by its manufacturer, is not guaranteed or endorsed by the publisher.
